# *Rabphilin* silencing causes dilated cardiomyopathy in a *Drosophila* model of nephrocyte damage

**DOI:** 10.1038/s41598-021-94710-7

**Published:** 2021-07-27

**Authors:** Estela Selma-Soriano, Carlos Casillas-Serra, Rubén Artero, Beatriz Llamusi, Juan Antonio Navarro, Josep Redón

**Affiliations:** 1 INCLIVA Biomedical Research Institute, 46010 Valencia, Spain; 2 Department of Genetics, University of Valencia, 46100 Burjassot, Spain; 3 Institute for Biotechnology and Biomedicine (BIOTECMED), University of Valencia, 46100 Burjassot, Spain; 4 CIPF-INCLIVA Joint Unit, Valencia, Spain; 5 Hypertension Unit, Hospital Clínico Universitario, 46010 Valencia, Spain; 6 CIBERObn, Health Institute Carlos III, Madrid, Spain

**Keywords:** Developmental biology, Genetics, Physiology

## Abstract

Heart failure (HF) and the development of chronic kidney disease (CKD) have a direct association. Both can be cause and consequence of the other. Many factors are known, such as diabetes or hypertension, which can lead to the appearance and/or development of these two conditions. However, it is suspected that other factors, namely genetic ones, may explain the differences in the manifestation and progression of HF and CKD among patients. One candidate factor is *Rph*, a gene expressed in the nervous and excretory system in mammals and *Drosophila,* encoding a Rab small GTPase family effector protein implicated in vesicular trafficking. We found that *Rph* is expressed in the *Drosophila* heart, and the silencing of *Rph* gene expression in this organ had a strong impact in the organization of fibers and functional cardiac parameters. Specifically, we observed a significant increase in diastolic and systolic diameters of the heart tube, which is a phenotype that resembles dilated cardiomyopathy in humans. Importantly, we also show that silencing of *Rabphilin* (*Rph*) expression exclusively in the pericardial nephrocytes, which are part of the flies' excretory system, brings about a non-cell-autonomous effect on the *Drosophila* cardiac system. In summary, in this work, we demonstrate the importance of *Rph* in the fly cardiac system and how silencing *Rph* expression in nephrocytes affects the *Drosophila* cardiac system.

## Introduction

Association between heart failure (HF) and chronic kidney disease (CKD) is recognized in multiple epidemiological and clinical studies. Both can be cause and consequence of each other^1^ Although multiple factors are known to contribute to developing both HF and CKD, such as diabetes and hypertension^1,2^, there are still unknown factors that could explain differences in the progression of HF and/or CKD among patients. Both cardiomyocytes and podocytes, specialized cells of the heart and kidney, respectively, play a key role in maintaining these organs' functional integrity. Both need a proper membrane and vesicular trafficking to function adequately, for which Rab GTPase proteins and their effectors are crucial elements. Dysfunction of these proteins leads to cardiomyopathy^3^ or renal damage^4,5^. One of their effectors, *Rabphilin-3A (Rph-3A)*, is implicated in vesicle docking/fusion reactions^6^. In humans, the *Rph*-3A gene encodes a protein that regulates exo- and endocytosis^6^ in neurons and glomerular podocytes, and the Human Protein Atlas and UniProt database indicates that *Rph-3A* is expressed in the human heart.

In podocytes, Rph-3A is found around vesicles contained in the foot-processes^4^. The NH2-terminal part of this protein interacts with α-actinin, a cytoskeletal protein, and promotes the formation of actin filaments providing a link between the synaptic vesicle and the cytoskeleton that is required for endocytosis^4,6,7^. The COOH-terminal part of Rph-3A contains two C2 domains that bind calcium and β-adduccin^6^ proteins, essential for podocyte integrity and homeostasis^4^.

*Rph-3A* exhibits altered expression in human proteinuric diseases and mouse models, suggesting a role for this gene in glomerulopathies^4^. Besides, it has been described that a polymorphism of this protein increases the risk of microalbuminuria in the general population ^8^. We have also shown previously that *Rabphilin* (*Rph*), the *Drosophila* ortholog of human *Rabphilin-3A*, participates in the endocytic pathway in nephrocytes and is necessary for the filtration of toxins from the hemolymph in this cell type. We also demonstrated that reduced levels of Rph caused structural alterations that had a negative impact on endocytosis and filtration rates^9^.

In the cardiac system, Rab proteins and their effectors are also implicated in cardiomyopathy. Specifically, Rabs 1, 4, and 6 are upregulated in a dilated cardiomyopathy mouse model and modulate cardiac myocyte growth^3,10,11^. Even though Rab proteins and the proteins that interact with them are expressed in cardiomyocytes and are related to heart problems that lead to heart failure^3^, the functions of the Rab GTPases and their effectors, such as Rph-3A, in the heart system are poorly understood.

*Drosophila* has an open circulatory system except for an approximately 1 mm of length contractile tube, which functions as a heart. Notably, the *Drosophila* heart has such developmental and functional homologies to the vertebrate heart that it has been used extensively to study human cardiac diseases^12–14^.

Given the relationship between nephrocytes and the heart in the development of metabolic diseases, we have studied the consequences on cardiac function of the silencing of *Rph* in nephrocytes or both in cardiomyocytes and nephrocytes. We discovered that knockdown of this gene in both tissues by *Hand-Gal4* leads to structural and functional changes that result in a decrease in survival. Of note, the impact that the RNA interference of *Rph* restricted to the nephrocytes has on cardiac function was less severe but still significant. Our data support a relevant role of *Rph* in both cardiomyocytes and nephrocytes in the maintenance of adequate cardiac function and support previous studies suggesting a functional link between nephrocyte function and cardiac failure^15–17^.

## Results

### *Rabphilin* is expressed in *Drosophila*’s heart

**Figure 1:**
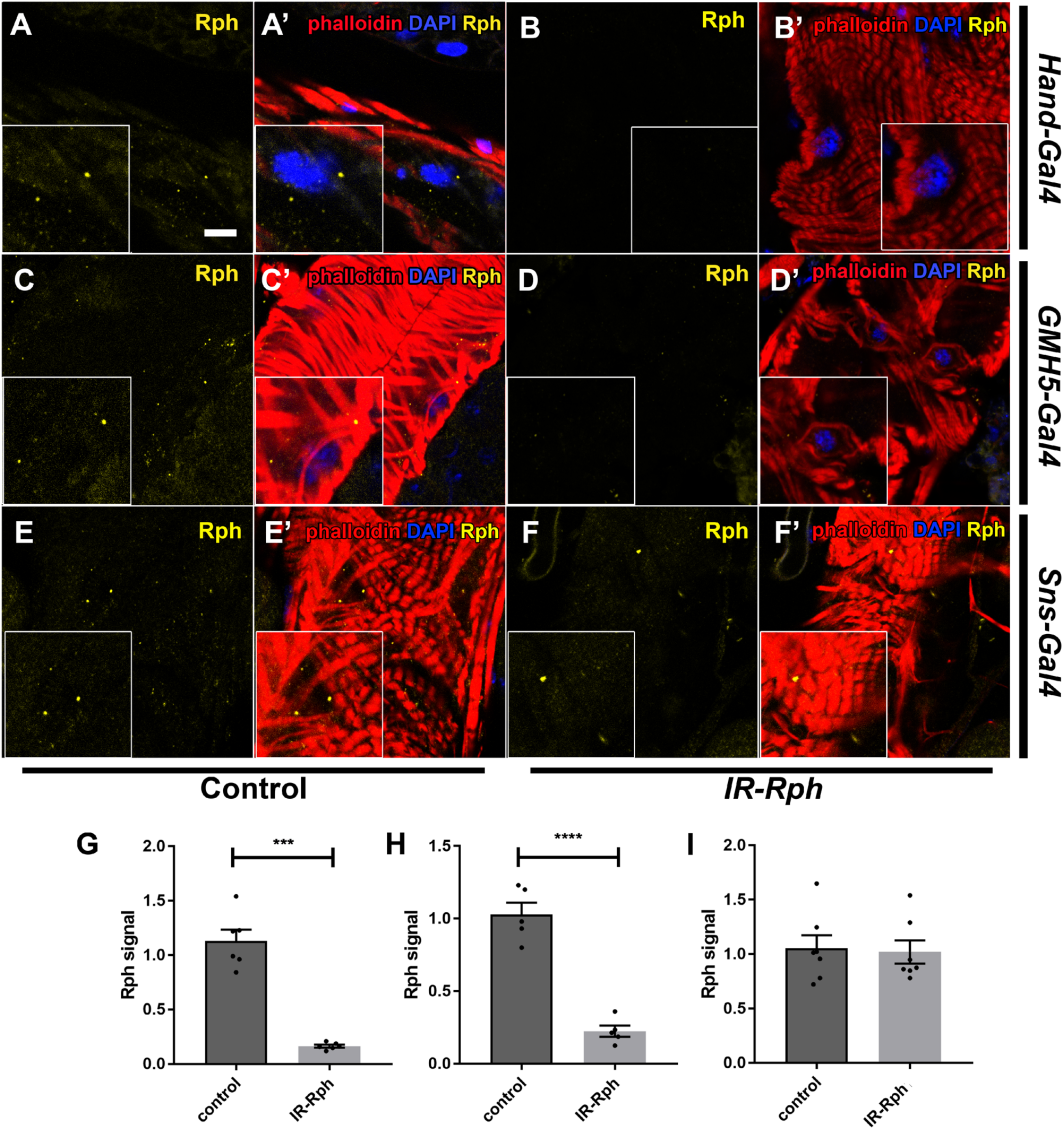
*Rabphilin* is expressed in adult *Drosophila* cardiomyocytes and Rph signal is decreased by expression of *UAS-IR-Rabphilin* line 1 construct. Representative confocal images of adult control (**A, A′, C, C′, E, E′**) and flies expressing *UAS-IR-Rabphilin* line 1 (*IR-Rph*, **B, B′, D, D′, F, F′**) under the control of the *Hand-Gal4* (**A, A′, B, B′**), *GMH5-Gal4* (**C, C′, D, D′**) and *Sns-Gal4* (**E, E′, F, F′**) driver. Immunostaining with the anti-*Rabphilin* antibody (in yellow) showed Rph presence in the heart of all control flies and *IR-Rph* flies driven by the *Sns-Gal4* line (**F, F′**). Rph signal was importantly reduced by the expression of the *Rph* interference construct line 1 in cardiomyocytes using *Hand-Gal4* and *GMH5-Gal4* drivers (**B, B′, D, D′**). Rph relative signal from flies expressing *IR-Rph* line 1 construct under *Hand-Gal4*, *GMH5-Gal4* and *Sns-Gal4* is shown in **G**, **H**, **I**, respectively. Nuclei were counterstained with DAPI (blue) and phalloidin (red) was used to stain actin filaments of the *Drosophila* heart. Images correspond to the A4 segment of the *Drosophila* abdomen and scale bar = 10 µm. Student’s t-test. ***p value < 0.001, ****p value < 0.0001.

**Figure 2:**
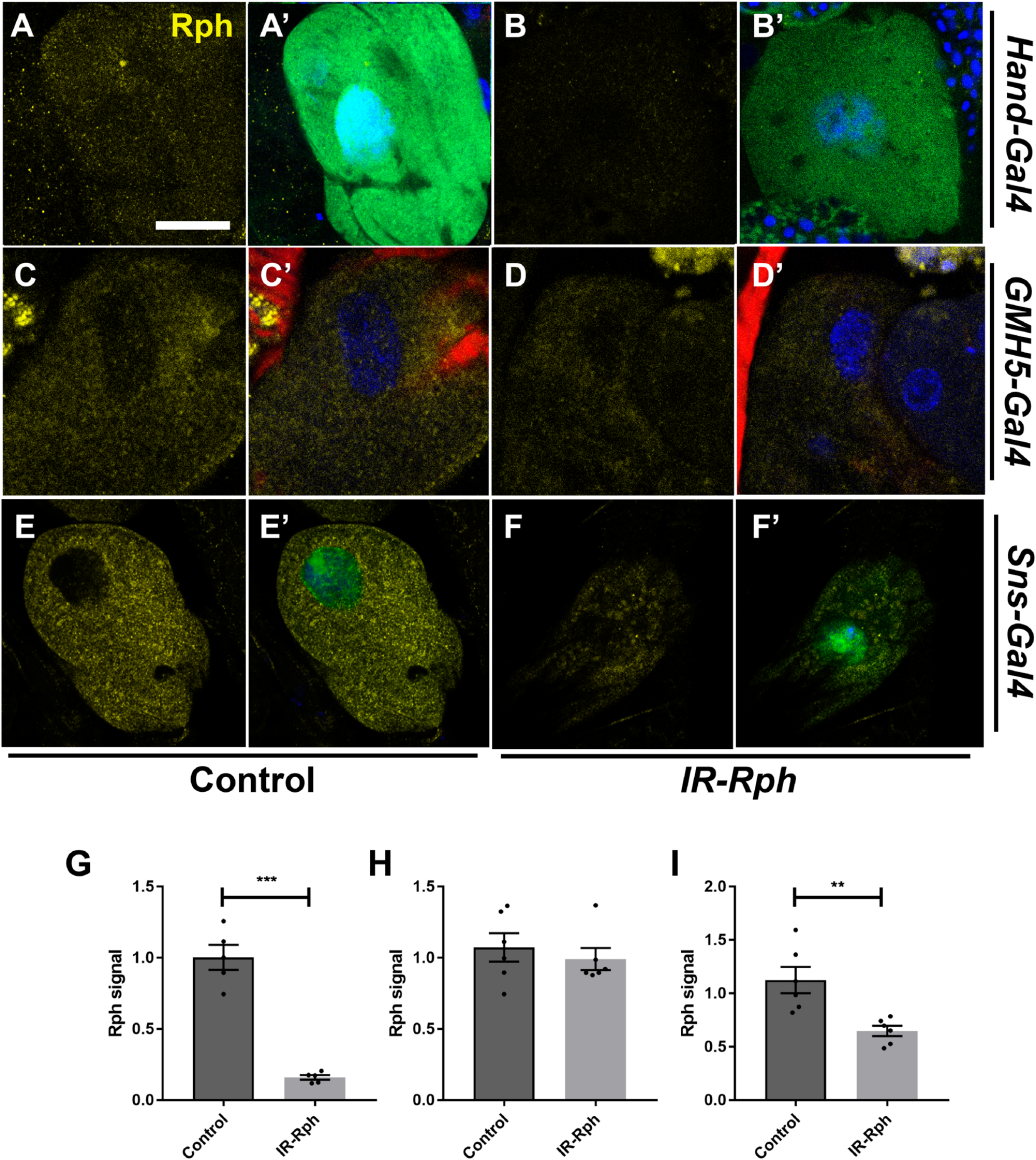
Rabphilin is expressed in adult Drosophila nephrocytes. Representative confocal images of adult control (**A, A′, C, C′, E, E**;* Hand-Gal4 UAS-GFP* >* yw*,* GMH5-Gal4 UAS-GFP* > *yw* and* Sns-Gal4* > *yw*, respectively) and flies expressing* UAS-IR-Rabphilin* line 1 (*IR-Rph*, **B, B′, D, D′, F, F′**) under the control of the *Hand-Gal4* (**A, A′, B, B′**), *GMH5-Gal4* (**C, C′, D, D′**) and *Sns-Gal4* (**E, E′, F, F′**) driver. Immunostaining with the anti-Rabphilin antibody (in yellow) showed Rph presence in pericardial nephrocytes of all control flies. Rph relative signal from flies expressing *IR-Rph* line 1 construct under* Hand-Gal4, GMH5-Gal4* and *Sns-Gal4* are represented in **G, H, I**, respectively. Nuclei were counterstained with DAPI (blue) and phalloidin (red) was used to stain actin filaments of the *Drosophila* heart surrounding pericardial nephrocytes. Scale bar = 10 µm. Student’s t-test. **p value < 0.01, ***p value < 0.001.

An immunofluorescence assay with the human anti-Rabphilin antibody was performed with heart tubes of control flies to check the presence of Rph in this tissue. The *UAS-Gal4* system was used to direct the silencing of *Rph* gene, with two different *UAS-IR-Rabphilin* lines, only in cardiomyocytes (*GMH5-Gal4* > *UAS-IR-Rabphilin* in Fig. 1 and Supplementary Figure 1C–D′) and both cardiomyocytes and nephrocytes (*Hand-Gal4*^18^ > *UAS-IR-Rabphilin* in Fig. 1 and Supplementary Figure 1A–B′). Previously, we observed *Rph* expression in nephrocytes^9^, and in this study, we report that *Rph* is also expressed in the *Drosophila* heart tube with a punctate pattern in the cytoplasm of cardiomyocytes (Fig. 1 and Supplementary Figure 1A, A′, C, C′, E and E′). To demonstrate the specificity of the antibody, the same assay was performed in flies with combined *Rph* RNAi, with two different lines of *Rph* RNAi constructs, in cardiomyocytes and nephrocytes (*Hand-Gal4 UAS-GFP* > *UAS-IR-Rabphilin),* and an important reduction of *Rph* expression was observed both in the cardiac tube and in nephrocytes (Fig. 1A–B′, G, Supplementary Figure 1A–B′, G, and Fig. 2A–B′, G). Furthermore, interference of *Rph* expression exclusively to cardiomyocytes using the specific driver *GMH5-Gal4*^19^ (*GMH5-Gal4 UAS-GFP* > *UAS-IR-Rabphilin*) showed decreased levels of Rph protein only in heart tissue but not in nephrocytes (Fig. 1 and Supplementary Figure 1C–D′, H and Fig. 2C–D′, H). Finally, interference of *Rph* expression restricted to nephrocytes using the nephrocyte-specific driver *Sns-Gal4*^20^ *(Sns-Gal4 UAS-GFP* > *UAS-IR-Rabphilin)* did not alter Rph protein levels in the heart serving the reduced signal in pericardial nephrocytes as an internal control of the experiment (Fig. 1 and Supplementary Figure 1E–F′, I and Fig. 2E–F, I).

### *Rph* silencing in cardiomyocytes and nephrocytes promotes alterations in heart structure

**Figure 3:**
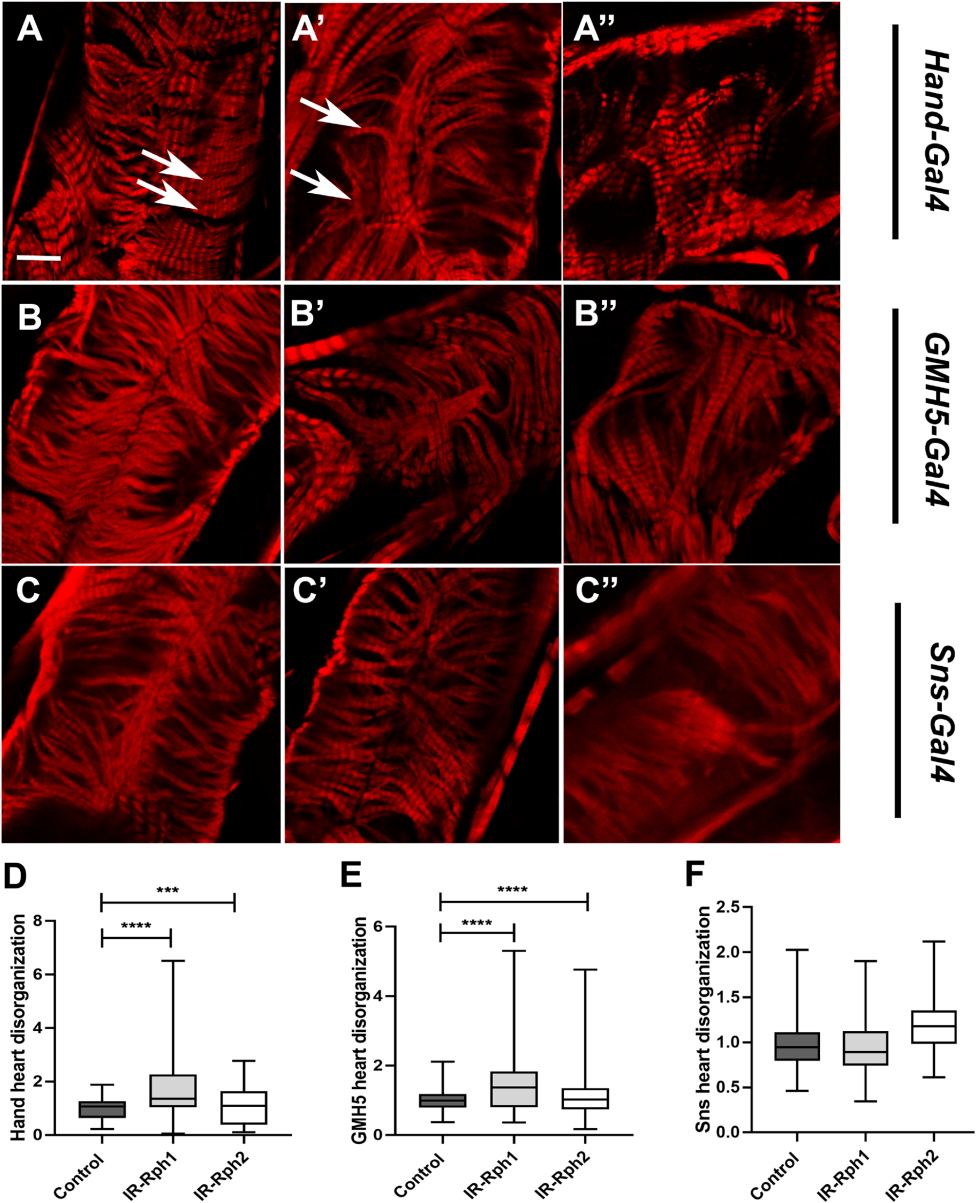
*Rph* interference in the heart promotes disruption of the disposition of circumferential myofibrils. Heart confocal images from the A1 segment (conical chamber) under the control of *Hand-Gal4* (**A–A″, D**), *GMH5-Gal4* (**B–B″, E**) or *Sns-Gal4* driver (**C–C″, F**). Phalloidin (red) stains actin fibers (arrow) in *Drosophila* heart tubes. The interference of *Rph* expression line 1, *IR-Rph1* (**B′, C′**) and interference of *Rph* line 2, *IR-Rph2* (**B″, C″**) in cardiomyocytes causes disorganization of actin fibers (arrow), but it does not affect flies with low Rph levels only in pericardial nephrocytes (**C–C″, F**). (**D**–**F**) Display the quantification of fibers disorganization. Genotypes of the control flies are *Hand-Gal4 UAS-GFP* > *yw, GMH5-Gal4 UAS-GFP* > *yw* and *Sns-Gal4 UAS-GFP* > *yw.* Scale bar = 10 µm. Bartlett’s test, ***p value < 0.001, ****p value < 0.0001.

We previously demonstrated alterations in the ultrastructure of nephrocytes upon the interference of *Rph* expression^9^ using the *Hand-Gal4* driver. As it is known that *Hand-Gal4* drives expression more strongly in cardiomy ocytes than in pericardial nephrocytes^15^, this driver was used to study the effect of *Rph* interference on *Drosophila* heart tube structure as well on pericardial nephrocytes. Moreover, to distinguish the plausible effect of nephrocytes using *Hand-Gal4* driver, we used *GMH5-Gal4,* which is a specific cardiomyocyte driver. Phalloidin staining was used to reveal the organization of the actin fibers in the *Drosophila* heart tube. In these preparations, we could detect significant disruption of the fiber organization in both cardiomyocyte driver’s lines (Fig. 3A–B″, Supplementary Figure 2), mainly abnormalities in the disposition of circumferential myofibrils, in flies expressing *UAS-IR-Rabphilin* line 1 (*Rph1*) in hearts (Fig. 3A′, B′, D, E and Supplementary Figure 2) as well as in flies expressing *UAS-IR-Rabphilin* line 2 (*Rph2)* (Fig. 3A″, B″, D, E). In contrast, interference of *Rph1* and *Rph2* expression restricted to nephrocytes, using *Sns-Gal4* driver, did not disorganize cardiac fiber (Fig. 3C, C′, C″, F), thus supporting an autonomous role of *Rph* in cardiac fiber organization.

### RNA interference-mediated silencing of *Rph* in cardiomyocytes and nephrocytes causes cardiomyopathy and lifespan reduction

**Figure 4:**
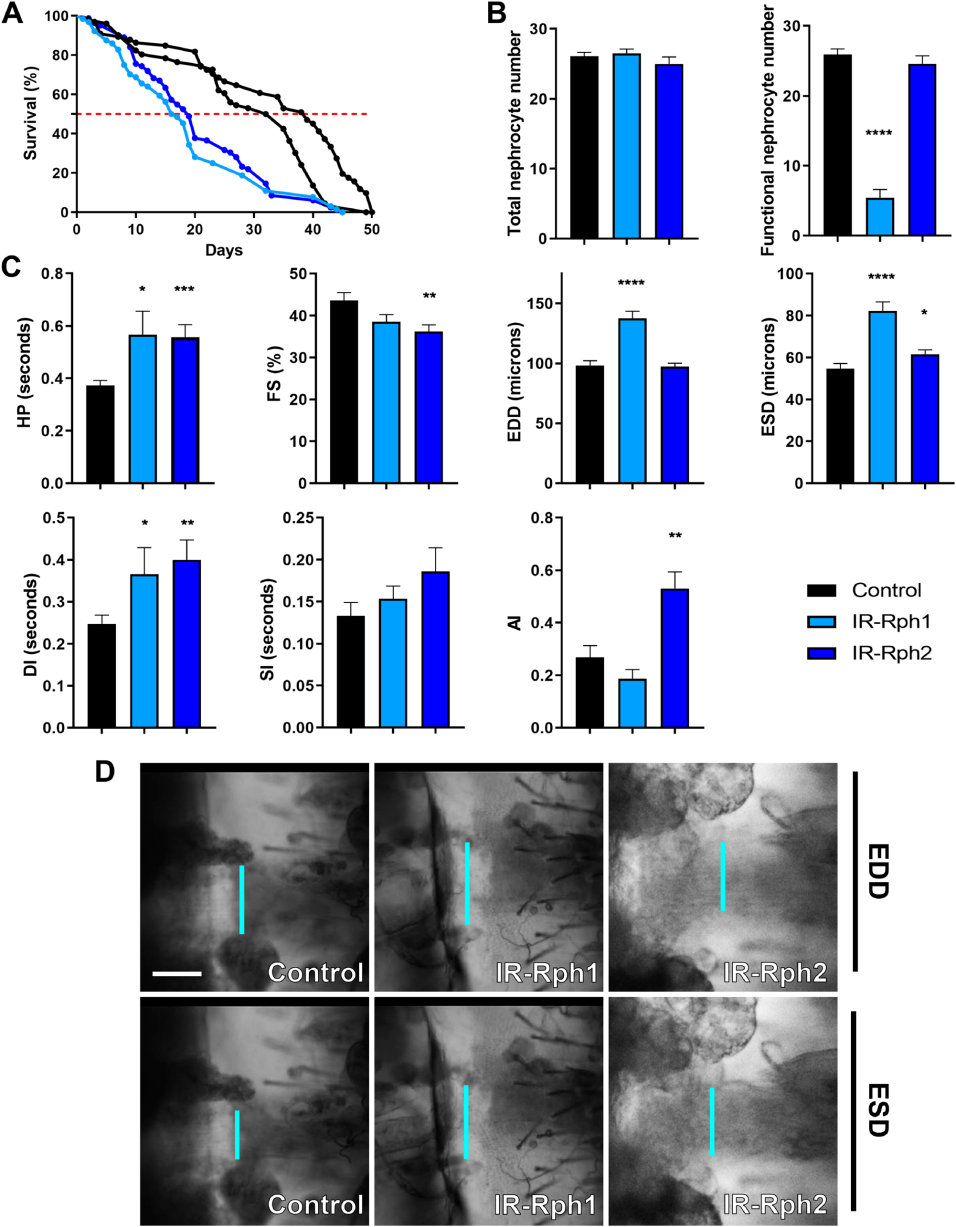
Cardiomyocyte and nephrocyte-specific silencing of *Rph* produces cardiac dysfunction in adult flies. (**A**) Survival curves of control (*Hand-Gal4 UAS-GFP* > *UAS-IR-bcdHand-Gal4 UAS-GFP* > *yw*, black lines) and *Rph* RNAi knockdown (blue lines) flies under the control of the *Hand-Gal4* driver. (**A**) The *Rph* silencing in both cardiomyocytes and nephrocytes impaired survival of adult *IR-Rph* flies. The horizontal red line marks the median survival. (**B**) Average number of total and functional pericardial nephrocytes in 1-week-old control (*Hand-Gal4 UAS-GFP* > *UAS-IR-bcd)* and *IR-Rph* flies (*Hand-Gal4 UAS-GFP* > *UAS-IR-Rph)*. (**C**) Adult heart function parameters represented as column bar graphs. (**D**) Representative micrographs of dissected fly abdomens showing heart tubes in the diastolic and systolic phases. Blue lines mark the distance between the heart walls in diastole and systole phases (EDD and ESD, respectively). The genotype of the control flies in panels C and D is *Hand-Gal4 UAS-GFP* > *yw.* Scale bar = 50 µm. Statistics results: log-rank (Mantel-Cox) test for survival: *p* value < 0.0001. Student’s t-test. *p value < 0.05, **p value < 0.01, ***p value < 0.001, ****p value < 0.0001.

The functional relevance of *Rph* expression in cardiomyocytes and nephrocytes was assessed by silencing the gene in these cell types with the *Hand-Gal4* driver and studying the survival curves and cardiac parameters in comparison to control flies. In adults, median survival was notably reduced from 33.5 and 39 days in control flies to only 16.5 days in *IR-Rph1* flies (*p* value < 0.0001) and to 19 days in *IR-Rph2* flies (*p* value < 0.0001) (Fig. 4A), with no significant differences between both *IR-Rph* lines. To account for this strong effect in median survival, and because the loss of nephrocytes is known to promote defects in cardiogenesis and cardiac function^21–23^, we counted the number of pericardial nephrocytes in 1-week-old adult females. The total average number of pericardial nephrocytes was the same for both genotypes, but when focusing on functional nephrocytes only, cells highly differentiated with strong capacity to endocytose and filtrate, which are characterized by *Hand*-driven GFP signal and intact nuclei as it is described in Selma-Soriano et al.^9^. The number of functional nephrocytes in *IR-Rph1* flies was significantly reduced compared to controls. This reduction was not detected using *IR-Rph2* flies, for which the number of functional nephrocytes did not significantly change compared to controls (Fig. 4B).

As we show, there is a strong impact on survival and functional nephrocyte number (Fig. 4A, B) induced by silencing *Rph* in both tissues: heart and nephrocytes. To elucidate if there is cardiac impairment, we studied cardiac parameters in 1-week-old adult female flies using SOHA software^24^ (Fig. 4C, D). In *Rph1* and *Rph2*-silenced flies, the heart period (HP) was significantly longer than control hearts (Fig. 4C). This originated from a significantly longer diastolic interval (DI), while the systolic interval (SI) remained the same (Fig. 4C). Diastolic and systolic diameters (EDD and ESD, respectively) were also greater than in controls in *IR-Rph* flies, but not in the case of EDD with *IR-Rph2* flies, which did not change compared to controls (Fig. 4C). The fractional shortening (FS) showed a trend towards reduction, not being significant in *IR-Rph1* flies but showing a significant reduction in *IR-Rph2* line (Fig. 4C). The arrhythmia index (AI), an indicator of the variability of the heart rhythm that is calculated by dividing the standard deviation of the heart period by its median, was unaltered in *IR-Rph1* flies, but it showed a significant increase in the *IR-Rph2* case (Fig. 4C). Taken together, these results indicate that *Rph* is necessary for the correct function of the *Drosophila* cardiac system.

### RNA interference-mediated silencing of *Rph* only in cardiomyocytes causes cardiomyopathy and lifespan reduction

**Figure 5:**
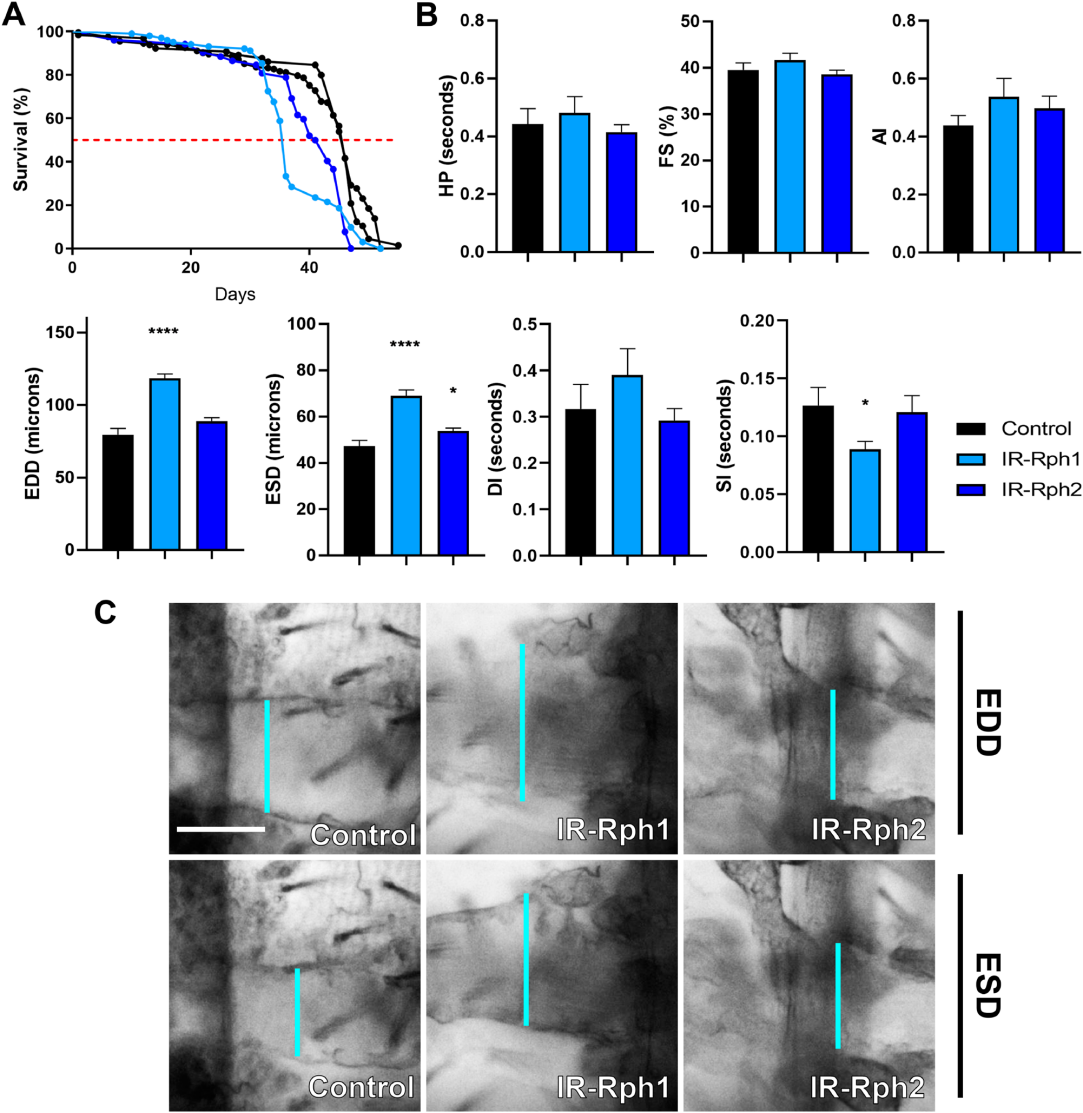
Cardiomyocyte-specific silencing of *Rph* produces cardiac dysfunction in adult flies. (**A**) Survival curves of control (*GMH5-Gal4 UAS-GFP* > *UAS-IR-bcd* and *GMH5-Gal4 UAS-GFP* > *yw* black lines) and *Rph* RNAi knockdown (blue lines) flies under the control of the *GMH5-Gal4* driver. (**A**) The *Rph* silencing in cardiomyocytes impaired survival of adult *IR-Rph* flies. The horizontal red line marks the median survival. (**B**) Adult heart function parameters represented as column bar graphs. (**C**) Representative micrographs of dissected fly abdomens showing heart tubes in the diastolic and systolic phases. Blue lines mark the distance between the heart walls in diastole and systole phases (EDD and ESD, respectively). The genotype of the control flies in **B**, **C** is *GMH5-Gal4 UAS-GFP* > *yw* Scale bar = 50 µm. Statistics results: log-rank (Mantel-Cox) test for survival: *p* value < 0.0001. Student’s t-test. *p value < 0.05, ****p value < 0.0001.

To check that Rph protein has an essential role in cardiac tissue, we performed the same experiment described in the previous section using a cardiomyocyte-specific driver, *GMH5-Gal4,* which promotes a reduction of Rph levels in the heart (Fig. 1 and Supplementary Figure 1C–D′). In adults, median survival was significantly reduced from 46 days in control flies to 36 days in *IR-Rph1* flies and to 42 days in *IR-Rph2* flies (*p* value < 0.0001) (Fig. 5A). As the driver did not affect the Rph levels in nephrocytes (Fig. 2), we did not analyse the total and functional nephrocytes number.

As we show in Fig. 5B, C, EDD and ESD values were significantly increased compared with control in *IR-Rph1* flies, but only ESD was higher in *IR-Rph2* flies. Notably, the systolic interval (SI) was decreased in the *IR-Rph1* line as compared with control, which could contribute to the reduction of median survival in this line (light blue line in Fig. 5A) as well as with *IR-Rph2* line (dark blue line in Fig. 5A). Taken together, these results indicate that *Rph* is necessary to maintain adequate cardiac diameters in *Drosophila’s* heart.

### *Rph* RNA interference in nephrocytes causes a slight extension of the diastolic diameter (EDD) and lifespan reduction

**Figure 6:**
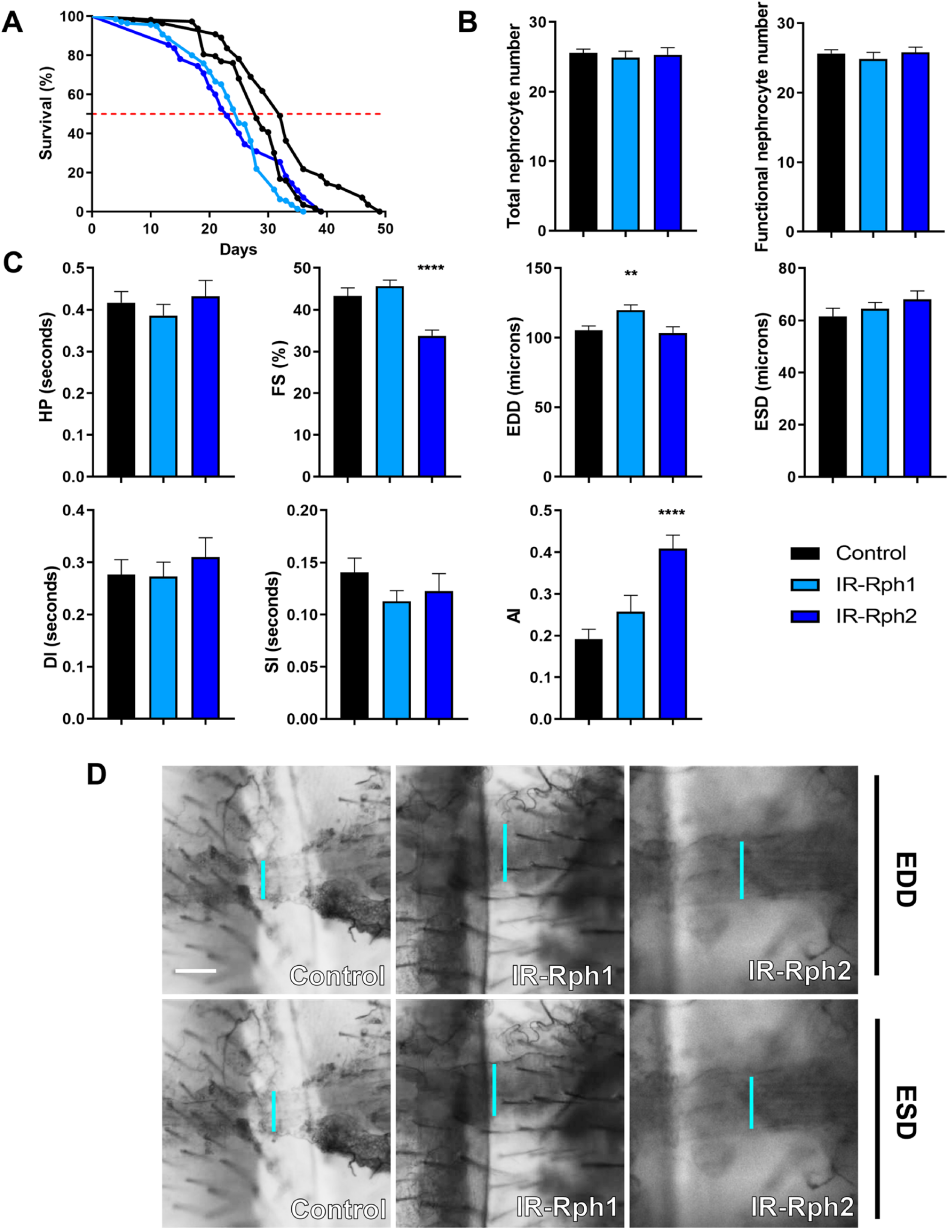
*Rph* RNA interference expression in pericardial nephrocytes produces a slight extension of the diastolic diameter in the adult *Drosophila* heart. (**A**) Survival curves of control (*Sns-Gal4 UAS-GFP* > *UAS-IR-bcd* and *Sns-Gal4 UAS-GFP* > *yw* black lines) and *Rph* RNAi knockdown (blue lines) flies under the control of the *Sns-Gal4* driver. (**A**) The *Rph* silencing in nephrocytes impaired survival of adult *IR-Rph* flies. The horizontal red line marks the median survival. (**B**) Average number of total and functional pericardial nephrocytes in 1-week-old control (*Sns-Gal4 UAS-GFP* > *UAS-IR-bcd)* and *IR-Rph* flies (*Sns-Gal4 UAS-GFP* > *UAS-IR-Rph)*. (**C**) Adult heart function parameters represented as column bar graphs. (**D**) Representative micrographs of dissected fly abdomens showing heart tubes in the diastolic and systolic phases. Blue lines mark the distance between the heart walls in diastole and systole phases (EDD and ESD, respectively). The genotype of the control flies in **C**, **D** is *Sns-Gal4 UAS-GFP* > *yw.* Scale bar = 60 µm. Statistics results: log-rank (Mantel–Cox) test for survival: *p* value < 0.0001. Student’s t-test. **p value < 0.01, ****p value < 0.0001.

Since nephrocytes have been reported to maintain normal cardiac function in flies^15–17^ we sought to test the hypothesis that silencing *Rph* exclusively in nephrocytes might originate cardiac dysfunction. This was assessed in adult flies expressing two different *Rph* interference constructs under the control of the *Sns-Gal4*^25^ and *Dot-Gal4*^26^ driver. *Rph* silencing under the control of *Dot-Gal4* was lethal at the pupa stage and, as a result, subsequent analyses could not be addressed. In flies with low Rph levels directed by *Sns-Gal4* driver, life span was slightly but significantly reduced (Fig. 6A, *p* value < 0.0001) compared to control flies; from 28 and 32 days in controls to 25 and 23 days for *IR-Rph1* and *IR-Rph2* lines, respectively (Fig. 6A); while the total and the average functional number of nephrocytes in 1-week-old adult females were the same for both genotypes (Fig. 6B).

Cardiac parameters in flies with nephrocyte-specific *Rph* RNAi knockdown revealed that the end-diastolic diameter (EDD) was significantly altered compared to controls in *IR-Rph1* flies (*p* value = 0.006, Fig. 6C, D) while the rest of the cardiac parameters analysed remained unchanged for this interference line (Fig. 6C, D). However, expressing *IR-Rph2* under the same driver’s control, fractional shortening (FS) and arrhythmia index (AI) were altered (Fig. 6C). The cardiac chamber's enlargement seemed to be a common feature of *Rph* RNA interference in both cardiomyocytes and nephrocytes, only in nephrocytes or only in cardiomyocytes. Thus, this data suggests that impaired nephrocyte function might have a non-autonomous or “at a distance” contribution to cardiac dysfunction as similarly seen in human chronic kidney disease patients^27–31^.

## Discussion

The adult *Drosophila* circulatory system comprises pairs of cardiomyocytes with circumferentially oriented contractile fibers; a non-cardiomyocyte-derived ventral longitudinal muscle located ventrally to the heart tube; and pericardial nephrocytes^32^. In flies, nephrocytes have been described as analogous to mammalian podocytes and also share the function to filter toxins and proteins from the hemolymph, equivalent to mammalian blood^33^. Nephrocytes express genes conserved in human renal podocytes and renal proximal tubule cells that are important for protein reabsorption and endocytosis in invertebrates and mammals. Specifically, we show that Rph, a well-conserved Rab effector protein, is expressed in the *Drosophila* heart. Similarly, we have previously reported *Rph* expression in pericardial nephrocytes^9^ and human podocytes^4^.

The relationship between proteinuria and cardiac dysfunction is well established in humans, but how nephrocytes influence cardiac function is unclear. This is an important question because of the potential to understand better the function of extracellular signals that affect cardiomyocyte biology, with potential direct implications for human diseases.

In *Drosophila*, it has been shown that pericardial nephrocytes can exert a paracrine effect on the cardiac system^15^. Although Das et al.^34^ described no changes in cardiac rate in flies without pericardial nephrocytes, it showed, as we report in our current work (Fig. 6), that they had a significantly reduced lifespan when compared to control flies. With this result, the authors suggested, as we do, that pericardial cells are important for the survival of adult flies. Although Das et al*.* do not detect any significant change in the heart beat parameter, it is important to consider that they ablate nephrocytes after embryonic development, while we achieve an interference in nephrocytes from embryonic stages^25^. In addition, recent work by Hartley et al.^16^ studied the impact of nephrocyte absence (due to the silencing of Klf15) on *Drosophila*’s heart. Although they did not study the lifespan and heart beat in flies lacking pericardial nephrocytes, Hartley et al. demonstrated some affected functional cardiac parameters, such as EDD or ESD, when nephrocytes were missing. Furthermore, other articles have associated loss of nephrocytes during development with defects in cardiogenesis^21–23^. Our study shows that combined RNA interference of *Rph* in cardiomyocytes and pericardial nephrocytes substantially impacts survival and originates cardiac alterations, including prolongation of diastolic interval and enlargement of cardiac chambers. The number of functional nephrocytes was also reduced in these flies. These deleterious effects were notably reduced when *Rph* RNA interference was restricted to nephrocytes, as only a small increase in diastolic diameter was detected, and the nephrocyte number was not altered (Fig. 6). As for the ESD values, using the *Sns-Gal4* driver, no changes are observed between the control and *IR-Rph* lines in contrast with the significant increases using the *Hand-Gal4* or *GMH5-Gal4* drivers, which could be explained by the different degrees of silencing achieved depending on the driver used. Importantly, although small, this alteration using the *Sns-Gal4* driver was enough to cause a significant reduction in lifespan. These data also support previous studies showing cardiac malfunction due to alterations in nephrocytes^15–17,21–23^.

Besides, our results indicate that the reduction of Rph levels only in cardiac tissue also impacted chamber diameter but not as severe as the phenotype observed when both tissues were affected, suggesting that nephrocyte malfunction impinges on the heart activity.

**Figure 7:**
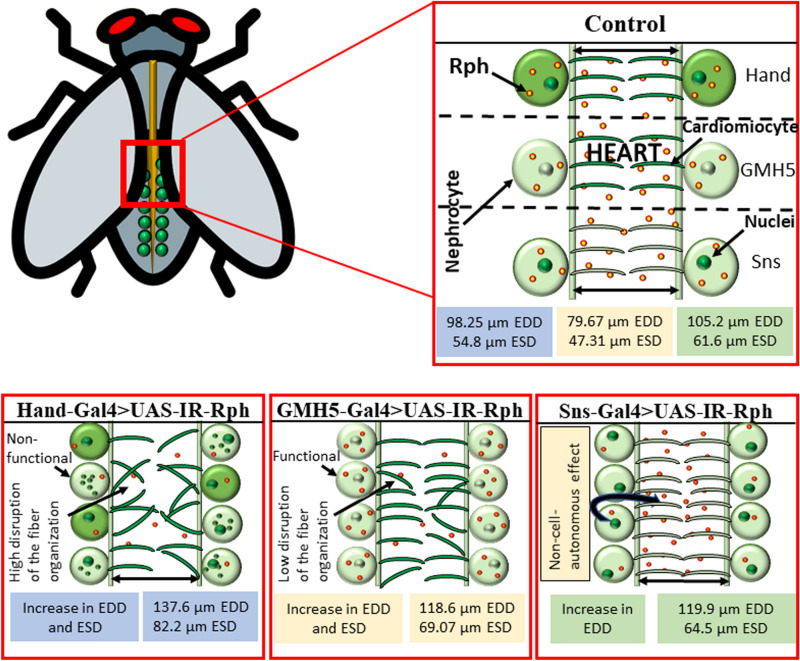
Graphical model of the cardiac alterations due to the silencing of *Rph* in heart and pericardial nephrocytes in *Drosophila*. RNA interference of *Rph* expression in the heart and nephrocytes (*Hand-Gal4* driver) and only in the heart (*GMH5-Gal4* driver) promotes an increase of EDD and ESD, disorganization of circumferential cardiac fibers, and a reduction in survival. Silencing of *Rph* expression in nephrocytes (*Sns-Gal4* driver) causes an increase in EDD, which reveals a paracrine effect of nephrocytes in the heart tube functioning. Text framed in blue, light red, and green indicates data coming from *Hand-Gal4*, *GMH5-Gal4,* and *Sns-Gal4* drivers, respectively. The big green dots surrounding the heart are the nephrocytes, and the smaller green dots inside represent the nuclei. The even smaller green dots that can be seen inside the nephrocytes when using *Hand-Gal4* driver represent the diffuse GFP signal characteristic of non-functional nephrocytes. The yellow/red dots represent Rabphilin inclusions. Finally, the transversal lines that are inside the heart represent the circumferential cardiac fibers. The intensity of the green colour indicates the tissue in which the corresponding *Gal4* construct drives the interference, being more intense in the places where it drives the interference most. Although in control flies we did not perform *Rph* interference, we did use the *Gal4* drivers and the same colour convention is employed to denote the expression patterns.

In the present study, we also demonstrated that interference of *Rph* expression in both tissues, as well as *Rph* silencing only in cardiomyocytes promotes actin fiber-disorganization. Interestingly, *Rph-3A* gene, the homolog of *Drosophila Rph*, binds the cytoskeletal protein actin and stimulates the reorganization of actin filaments^6,7^. Actin disorganization could be contributing to the increase in diastolic and systolic diameters that we observed in the flies with combined *IR-Rph* interference and knockdown *Rph Drosophila* cardiomyocytes. Accordingly, mutations in genes that encode components of the cytoskeleton, such as actin, genes that control the interaction of actin with other proteins, and other alterations in the cytoskeleton, have been previously associated with the appearance of dilated cardiomyopathy^35–37^, a disease that is associated with enlargement of cardiac chambers in patients. Of note, RNA interference of *Rph* exclusively in nephrocytes produced a slight increase of diastolic diameter without causing an evident alteration in actin organization, meaning that a non-cell-autonomous effect originated from nephrocytes might exert small modifications in the heart structure (Fig. 7).

Taken together, our work indicates a relevant role for *Rph* in both the heart and the nephrocytes suggesting a potential implication in the homeostasis between these two tissues, which supports that mutations or polymorphisms in this gene may be of biomedical relevance.

## Materials and methods

### *Drosophila* strains

*UAS-IR-Rabphilin* line 1 (referred to as *IR-Rph1*, BDSC stock number: 25950); *UAS-IR-bcd* and *yw* stocks were obtained from *Bloomington Drosophila Stock Center* (Indiana University); *UAS-IR-Rabphilin* line 2 (referred to as *IR-Rph2*, construct ID: 107492) was obtained from *Vienna Drosophila resource center*) and *Sns-Gal4 UAS-GFP* was obtained from Dr. M. Ruiz-Gómez (Centro de Biología Molecular Severo Ochoa, Madrid). *IR-Rph1* and *IR-Rph2* use different RNA interference approaches, and while the *IR-Rph1* construct generates a dsRNA, *IR-Rph2* generates a hairpin that is also processed endogenously. The recombinant line *Hand-Gal4 UAS-GFP* was generated in our group to mark adult nephrocytes and cardiomyocytes. The cardiomyocyte-specific driver *GMH5-Gal4 UAS-GFP* was kindly provided by Dr. Bodmer (Sanford Burham Institute, CA). All crosses were maintained at 25 ºC on standard nutritive medium.

### *Drosophila* lifespan analysis

More than 100 males per genotype were collected and placed in tubes containing standard nutritive medium and kept at 29 ºC to ensure maximal silencing of *Rph*. The number of deaths was scored on a daily basis, and flies were transferred to fresh medium every 2–3 days. Survival curves were obtained using the Kaplan–Meier method, and statistical curve comparisons were carried out according to the log-rank (Mantel-Cox) test (α = 0.05).

### Immunofluorescence staining

Adult hearts from 7-day-old females were dissected in PBS 1 X according to Selma-Soriano and Chakraborty^38^, fixed with 4% paraformaldehyde for 20 min and permeabilized by PBS containing 0.3% Triton-X (PBS-T) for 10 min, 3 times. Hearts were blocked in PBS-T containing 0.5% BSA for 30 min at room temperature and incubated with human anti-Rabphilin (1:200) (Abcam, ab3338). After 3 washes with PBS-T, the AlexaFluor-647 donkey anti-rabbit (1:1000) (Life Technologies, A31573) was incubated for 2 h at room temperature. The images were taken with an LSM 800 confocal microscope (Zeiss) using 40 × oil objective.

### Rph signal quantification

ZEN software was used to quantify the Rph signal from immunofluorescent images. The heart and nephrocyte signal areas were selected, and the intensity and frequency of the pixels were scored. For the analysis, at least three different biological samples were used. Results were analysed using a two-tailed unpaired Student's t-test (α = 0.05), applying Welch's correction whenever necessary.

### Actin disorganization analyses

Quantification of the disorganization of the myofibrils in the cardiomyocytes was performed with Voronoi’s Diagrams^39–41^. Briefly, Voronoi’s diagrams are a geometrical construction that allows a tessellation of Euclidean plane. Given a set of points on a Euclidean plane, perpendicular bisectors among these points are generated, giving rise to a set of polygons and being their perimeters equidistant to their closest points on the plane. The cardiac actin fibers, fixed following Ca^2+^ chelation (10 mM EGTA) to stop heart beating at the same phase and stained with phalloidin, were outlined using ImageJ software and Voronoi’s areas were generated (Supplementary Figure 2). In organized hearts, the areas obtained were similar and had no variance among them. In disorganized hearts, where the circumferential fibers did not keep the same distances among them and had more convoluted paths than organized hearts, the areas generated were more different, thus implying a higher variance value. To test this statistically, we performed a Bartlett test to check the homogeneity of variance among genotypes (α = 0.05).

### Cardiac analyses

1-week-old female hearts were dissected as previously described in Chakraborty^42^. For the recording, a Leica microscope with an ORCA Flash (Hamamatsu) high-speed digital camera was used to take 20 s recordings at a minimum speed of 150 frames/s. Different cardiac parameters were measured using SOHA software^24^. Results were analysed by two-tailed non-paired Student’s t-test (α = 0.05).

### Phalloidin staining

The semi-intact heart preparations were dissected in PBS 1 X. The hearts were incubated with phalloidin (1:1000 in PBT, #P1951, Sigma) for 20 min. Samples were mounted in Vectashield (Vector). All confocal images were taken with an LSM 800 confocal microscope (Zeiss) using a 40 × oil objective.

### Quantification of nephrocytes number

For the analysis of the number of nephrocytes, 1-week-old adult female fly hearts were dissected in 1 × PBS. Images were obtained with a Leica DM4000 B LED microscope using a 10 × or 20 × air objective. Functional nephrocytes were identified as cells with intact nuclei and a strong GFP signal level.

## Supplementary Information

Supplementary Information.
