# Supplementary material for: Rabphilin silencing causes dilated cardiomyopathy in a Drosophila model of nephrocyte damage
Source: Sci Rep. 2021 Jul 27;11:15287. doi: 10.1038/s41598-021-94710-7 (PMC8316431; doi:10.1038/s41598-021-94710-7)
Supplement: Supplementary file 1 — Supplementary Information. [file 41598_2021_94710_MOESM1_ESM.docx]

**SUPPLEMENTARY**

**
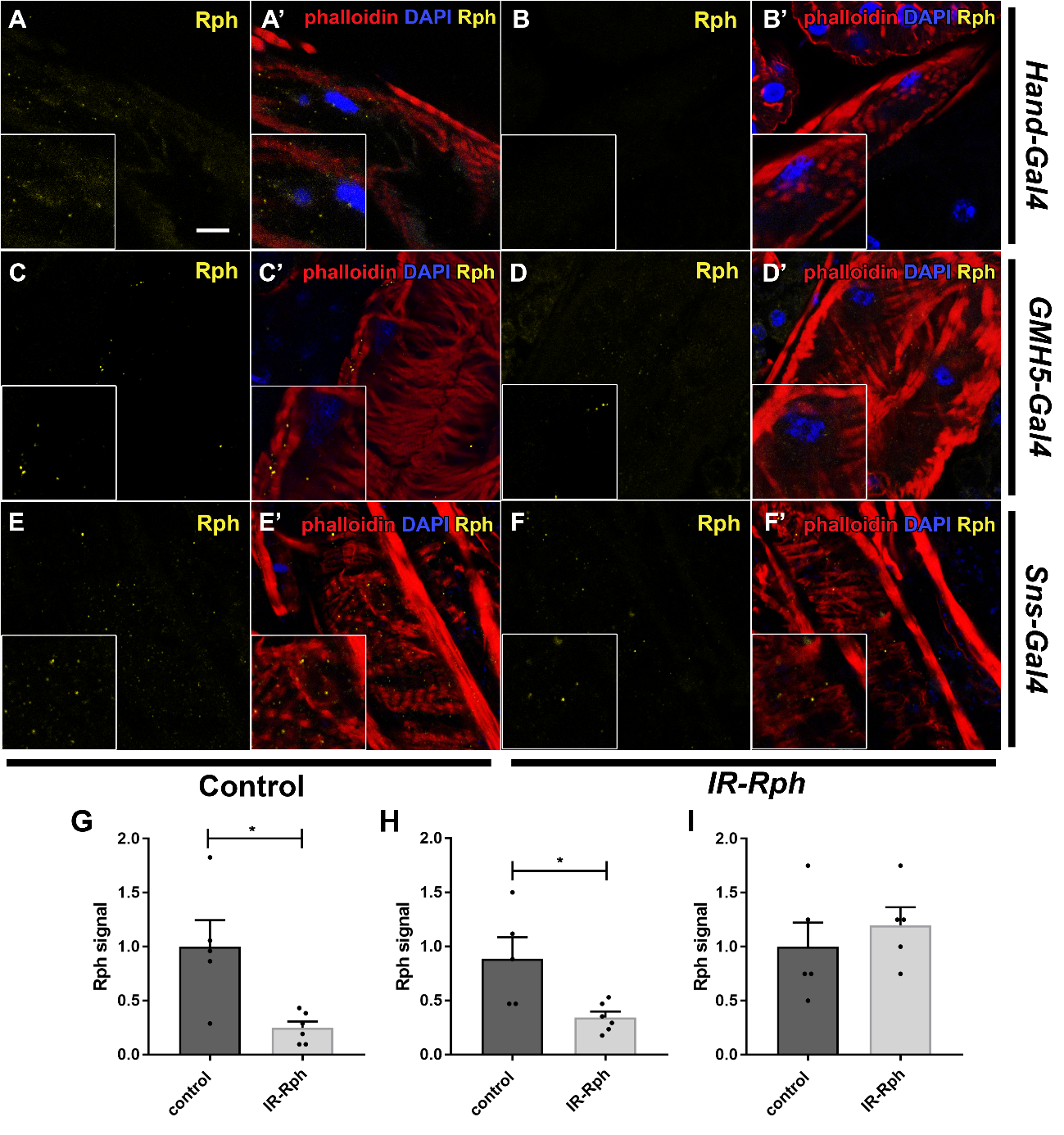
**

**Supplementary Figure 1. *Rabphilin* is expressed in adult *Drosophila* cardiomyocytes and Rph signal is decreased by expression of *UAS-IR-Rabphilin* line 2 construct.** Representative confocal images of adult control (A, A’, C, C’, E and E’) and flies expressing *UAS-IR-Rabphilin* line 2 (*IR-Rph*, B, B’, D, D’, F and F’) under the control of the *Hand-Gal4* (A, A’, B and B’), *GMH5-Gal4* (C, C’, D and D’) and *Sns-Gal4* (E, E’, F and F’) driver. Immunostaining with the anti-*Rabphilin* antibody (in yellow) showed Rph presence in the heart of all control flies and *IR-Rph* flies driven by the *Sns-Gal4* line (F and F’). Rph signal was importantly reduced by the expression of the *Rph* interference construct line 2 in cardiomyocytes using *Hand-Gal4* and *GMH5-Gal4* drivers (B, B’, D and D’). Rph relative signal from flies expressing *IR-Rph* line 2 construct under *Hand-Gal4*, *GMH5-Gal4* and *Sns-Gal4* are shown in panels G, H and I, respectively. Nuclei were counterstained with DAPI (blue) and phalloidin (red) was used to stain actin filaments of the *Drosophila* heart. Genotypes of the control flies are *Hand-Gal4 UAS-GFP > yw, GMH5-Gal4 UAS-GFP > yw* and *Sns-Gal4 UAS-GFP > yw.* Images correspond to the A4 segment of the *Drosophila* abdomen. Scale bar= 10 µm. Student’s t-test. *p-value < 0.05.


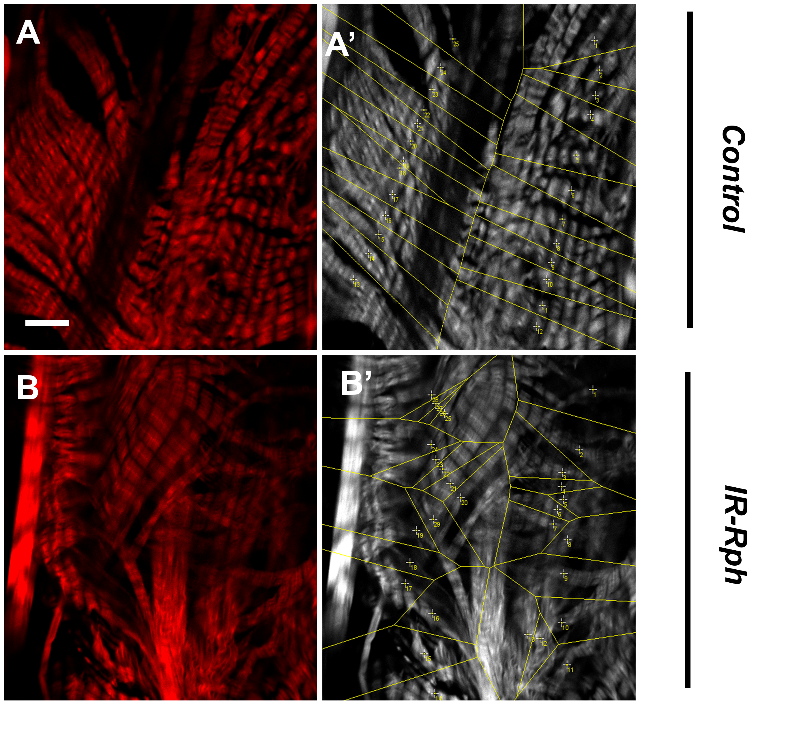


**Supplementary Figure 2. Quantification of cardiac actin fibers disorganization using Voronoi’s diagrams**. Confocal images of *Drosophila*’s heart under the control of *Hand-Gal4* driver in control (A) and *IR-Rph1* (B) flies. Phalloidin (red) stains actin fibers in *Drosophila* heart tubes. Several points are placed on the circumferential actin fibers. Once the points are placed, Voronoi areas are obtained (yellow lines) using its corresponding plug-in on ImageJ software (A’ and B’). All images were taken with the same settings. Scale bar= 10 µm.
